# Diffusion-weighted magnetic resonance imaging as an early prognostic marker of chemoradiotherapy response in squamous cell carcinoma of the anus: An individual patient data *meta*-analysis

**DOI:** 10.1016/j.phro.2024.100618

**Published:** 2024-07-31

**Authors:** Bettina A. Hanekamp, Pradeep S. Virdee, Vicky Goh, Michael Jones, Rasmus Hvass Hansen, Helle Hjorth Johannesen, Anselm Schulz, Eva Serup-Hansen, Marianne G. Guren, Rebecca Muirhead

**Affiliations:** a Department of Radiology, Oslo University Hospital, Oslo, Norway; b Institute of Clinical Medicine, University of Oslo, Oslo, Norway; c Nuffield Department of Primary Care Health Sciences, University of Oxford, Oxford, UK; d Cancer Imaging, School of Biomedical Engineering & Imaging Sciences, King’s College London, London, UK; e Department of Radiology, Guy’s and St Thomas’ NHS Foundation Trust, London, UK; f University of Newcastle, Callaghan, Australia; g Section for Radiation Therapy, Department of Oncology, Copenhagen University Hospital, Rigs-hospitalet, Copenhagen, Denmark; h Department of Clinical Physiology and Nuclear Medicin, Copenhagen University Hospital, Rigs-hospitalet, Copenhagen, Denmark; i Department of Oncology, Copenhagen University Hospital, Herlev and Gentofte, Denmark; j Department of Oncology, Oslo University Hospital, Oslo, Norway; k Oxford University Hospital NHS Foundation Trust, Oxford, UK

**Keywords:** Anal cancer, Diffusion Weighted Magnetic Resonance Imaging, Apparent diffusion coefficient, Biomarker, Response assessment, Prognostic

## Abstract

**Background and purpose:**

Squamous cell carcinoma of the anus (SCCA) can recur after chemoradiotherapy (CRT). Early prediction of treatment response is crucial for individualising treatment. Existing data on radiological biomarkers is limited and contradictory. We performed an individual patient data *meta*-analysis (IPM) of four prospective trials investigating whether diffusion-weighted (DW) magnetic resonance imaging (MRI) in weeks two to three of CRT predicts treatment failure in SCCA.

**Material and methods:**

Individual patient data from four trials, including paired DW-MRI at baseline and during CRT, were combined into one dataset. The association between ADC volume histogram parameters and treatment failure (locoregional and any failure) was assessed using logistic regression. Pre-defined analysis included categorising patients into a change in the mean ADC of the delineated tumour volume above and below 20%.

**Results:**

The study found that among all included 142 patients, 11.3 % (n = 16) had a locoregional treatment failure. An ADC mean change of <20 % and >20 % resulted in a locoregional failure rate of 16.7 % and 8.0 %, respectively. However, no other ADC-based histogram parameter was associated with locoregional or any treatment failure.

**Conclusions:**

DW-MRI standard parameters, as an isolated biomarker, were not found to be associated with increased odds of treatment failure in SCCA in this IPM. Radiological biomarker investigations involve multiple steps and can result in heterogeneous data. In future, it is crucial to include radiological biomarkers in large prospective trials to minimize heterogeneity and maximize learning.

## Highlights

• ADC parameters during treatment were not associated with outcome in anal cancer.
• Individual patient *meta*-analysis was useful to increase patient numbers and events.
• Radiological biomarkers must be integrated into large anal cancer trials.

## 1 Introduction

Squamous cell carcinoma of the anal canal (SCCA) is a rare cancer with a low but increasing incidence rate of around 1 per 100.000 in Western countries [1]. The standard curative treatment of localised SCCA is chemoradiotherapy (CRT) with concomitant mitomycin C (MMC) and 5-fluorouracil (5-FU) or capecitabine [2], [3]. Although disease-free survival (DFS) is reported as 73 % for patients with SCCA after treatment with CRT, for patients with locally advanced disease, DFS is as low as 63 % [2]. The identification of early prognostic biomarkers would offer the potential to personalise treatment and improve outcomes.

Prior to treatment, magnetic resonance imaging (MRI) is routinely used for staging of SCCA, [3], [4], [5], [6]. Diffusion-weighted MRI (DW-MRI) enables quantification of the motion of water molecules. In malignant tissues, the movement of water molecules is restricted, resulting in a lower apparent diffusion coefficient (ADC) than in healthy tissues. Within oncology, the use of DW-MRI includes the identification of malignant tumour sites, characterisation of tumour aggressiveness, and response evaluation after treatment, and investigation is ongoing into its use as an imaging-based biomarker [7].

In squamous cell carcinomas of other tumour sites, DW-MRI ADC has been investigated as a prognostic biomarker with mixed results [8], [9]. In both head and neck and cervical cancer, the change in ADC three weeks into radiotherapy has consistently shown a correlation with cancer outcomes [10], [11], [12]. In anal cancer, three published studies evaluated the correlation between DW-MRI before and during CRT with treatment failure. All three trials included 20–60 patients; they investigated many different parameters and reported slightly different results, each generating different hypotheses [13], [14], [15]. A recurring problem with radiological biomarkers studies is small patient numbers. Particularly in rare tumours such as anal cancer, the number of patients available to recruit to studies is small. In addition, the relatively good outcomes make the number of events low. These factors make it unlikely these retrospective single-centre studies will ever have the numbers required to reach robust statistical conclusions. [16].

To address these issues, we aimed to investigate the correlation between any parameter from paired DW-MRI at baseline and during weeks two to three of treatment, with locoregional or any treatment failure, using an individual patient data *meta*-analysis of the three published studies and a further completed but unpublished study.

## 2 Materials and methods

We analysed four prospective trials, acquiring data between 2011 and 2017, in patients receiving radical CRT for SCCA who had paired DW-MRI before treatment (baseline) and week two to three of treatment (mid-treatment); the UK ART study (ClinicalTrials.gov: NCT02145416), Australian study (ACTRN12614001219673), Norwegian study (NCT01937780), and Danish study (NCT01330186). We performed the analysis in line with the Preferred Reporting Items for Systematic Review and Meta-Analyses (Individual Patient Data) (PRISMA-IPA) [17].

### 2.1 Search strategy

We searched PubMed, Medline (OVID version), Embase (OVID version), the Cochrane Library and clinicaltrials.gov from their inception until 16th October 2023 for additional trials. Search terms were “anal” AND “Diffusion-Weighted MRI” and all relevant keyword variations. In addition to the three published studies [13], [14], [15], [18], one Danish study (NCT01330186) was identified, investigators were approached, and the results were added to the *meta*-analysis. A further Canadian trial was initially registered but then withdrawn, and no associated results were identified (NCT01053923).

### 2.2 Patients

Eligibility was broadly similar between the trials, incorporating patients with histologically confirmed SCCA, without metastasis, who were planned for radical CRT and able to undergo serial MRI scans. Details on eligibility, treatment and follow-up have previously been reported for the UK ART study, Australian study, and Norwegian study [13], [14], [15], [18], [19] with the Danish study using identical eligibility and comparable treatment and follow-up schedules.

### 2.3 Image acquisition and analyses

All included centres accomplished paired MRI scans prior to treatment and mid-treatment. In the UK ART, Australian, and Norwegian study, the mid-treatment scan was taken in week two of CRT and in the Danish study, it was taken in week three. MRI scans, including DW and T2W sequences, were performed on a 3 T MR scanner in the UK ART, Australian, and Norwegian study; in the Danish study, a 1 T MR scanner was used. Slightly different DW imaging parameters and combinations of b-values ranging from b 0 to b 1500 were used at the four centres (Table 1). In all four centres, a volume of interest (VOI) was created around the tumour and any involved lymph nodes >2 cm on T2 weighted images as detailed in consensus recommendations [7]. Subsequently, all volumes were propagated to DW images. The ADC parameters were extracted from ADC volume histogram analysis. The tumour volume (VOI) delineation and ADC histogram analyses were performed locally at the four centres.

**Table 1:** Magnetic field strength and Diffusion Weighted (DW) imaging parameters. Tesla (T); Magnetic Resonance Imaging (MRI).

Parameter	UK ART	Norway	Denmark	Australia
Magnetic field strength (T) of the MRI machine	3	3	1	3
*b-*values (s/mm^2^)	0, and 1500	0, and 1200	50,100,150, 500,800, and 1000	0,400,800, and 1200
Repetition time (ms)	≥3300	2167	5000	6200
Echo time (ms)	58.2	75	80	90
Slice thickness (mm)	3	5	4	3.5
Slice gap (mm)	0.3	1	0	3.5
Matrix size	256 × 256	116 × 132	116 × 130	170 × 170
No. averages	8	4	4	6
Field of view (mm^2^)	400 × 400	400 × 279	230 × 262	230 × 230
No. slices	21	35	Variable	26

### 2.4 Data collection

Individual patient data included sex, age, TNM stage, CRT details, and outcomes. The following MRI parameters for the scans at baseline and mid-treatment were collected: T2 volume of the gross tumour volume (GTV) or VOI, which are interchangeable in this study, which was either primary macroscopic tumour or involved node >2 cm. The VOI included areas of necrosis in all studies as they could not be reliably removed. The maximum and the mean ADC value within the delineated VOI (ADCmax; ADCmean), and if available, skewness, kurtosis, and standard deviation were created from the ADC volume histogram. The software used for the ADC histogram in the individual centres were: in Australia, QIPCM Temporal Dynamic Analysis (TDA) software (University Health Network (UHN), Toronto, Canada); in the UK, Eclipse software (version 13.0, Varian Medical Systems, Palo Alto, CA, USA); in Norway, LIFEx freeware for radiomic feature calculation (Paris, France); in Denmark, Nordic i*c*e software package (Bergen, Norway). Further details on ADC calculations are given in the individual publications [13], [14], [15]. All individual patient data were anonymised by their study investigators and provided to the University of Oxford, UK, for combination and analysis.

### 2.5 Outcomes

Any treatment failure was defined as a combination of three internationally defined outcomes: local, regional, or distant failure [20]. Locoregional failure was defined as a failure to demonstrate a complete response (CR) 6 months after CRT or evidence of disease within the radiotherapy field after CR had been achieved. Distant failure was the development of metastatic disease.

### 2.6 Statistical analysis

A one-stage *meta*-analysis was performed due to the small sample size and number of events per study, as recommended by Riley *et al.* [21], where data from the four trials was treated as one large dataset and analyses were performed for the overall cohort and adjusted for the study.

A preplanned statistical analysis plan was designed at outset in keeping with PRIMA-IPD guidance. To explore the relationship between ADC volume histogram parameters and clinical outcomes, logistic regression was used to derive odds ratios (95 % confidence intervals (CI)) for the association between MRI parameters at baseline (scan 1) and mid-treatment (scan 2) and the percentage change in parameters between scans with locoregional or any failure. Each logistic model included a stratified intercept for the study (a study indicator for each survey separately) to adjust for differences between the four studies. The area under the curve (AUC) was derived for each parameter to assess how well parameters perform in identifying treatment failure. Analyses were performed in Stata SE V17.0.

Based on previous findings in small studies on SCC of other body regions [9], [11], [22], the primary endpoint of the UK ART study [14] was the number/proportion of patients who had a percentage change in ADCmean between the two scans (ΔADCmean) of <20 %. We presented the number and proportion of patients in the *meta*-analysis with ΔADCmean <20 % with data on the number of those who failed treatment for both locoregional and any failure separately.

Considering potential measurement inaccuracy due to small tumour size or including nodes >2 cm, the above analysis was repeated on the subgroup of patients with primary tumours >5 cm.

We investigated the correlation between ADCmean and the gross tumour volume (GTV) size over the course of radiotherapy using a Spearman correlation. This analysis was performed using data from the UK ART study, the Norwegian study, and the Danish study only. While the GTV was delineated in the Australian dataset, the volume was not extracted. Due to the time since the primary analysis, it was impossible to perform this retrospectively.

Two sensitivity analyses were planned. The first was to use mixed-effects logistic regression to account for repeated ADC measurements for patients with additional nodes. However, due to the small sample size and number of events overall, mixed-effects models did not converge, so this analysis was impossible. The second was to use a two-stage *meta*-analysis approach, which is likely underpowered because each small study is initially analysed separately.

## 3 Results

There were 142 patients with 150 volumes of interest (VOI) (primary tumour and lymph nodes >2 cm) included in the *meta*-analysis. The number of patients and VOI from each study were: UK ART study, 23 patients with 26 VOIs in a single centre; Australian study, 20 patients with 20 VOIs in 3 centres; Norwegian study, 39 patients with 44 VOIs in a single centre; Danish study, 60 patients with 60 VOIs in a single centre. Patient characteristics at baseline are provided in Table 2*.* One patient in the Norwegian study had a paraaortic (M1) lymph node included in the radiation field.

**Table 2:** Patient characteristics at baseline. Gross tumour volume (GTV).

Characteristic	UK (n = 23)	Norway (n = 39)	Australia (n = 20)	Denmark (n = 60)	Combined (n = 142)
*Age at enrolment (years)*					
Mean (SD)	60.3 (11.0)	59.5 (11.1)	58.0 (8.9)	59.9 (10.0)	59.6 (10.3)
Median (min–max)	60.0 (36.0–82.0)	59.0 (40.0–90.0)	58.5 (38.0–77.0)	60.7 (38.5–90.8)	60.0 (36.0–90.8)
*Sex*					
Male	8.7 % (n = 2)	25.6 % (n = 10)	15.0 % (n = 3)	25.0 % (n = 15)	21.1 % (n = 30)
Female	91.3 % (n = 21)	74.4 % (n = 29)	85.0 % (n = 17)	75.0 % (n = 45)	78.9 % (n = 112)
*T stage*					
T1	−	2.6 % (n = 1)	5.0 % (n = 1)	3.3 % (n = 2)	2.8 % (n = 4)
T2	60.9 % (n = 14)	51.3 % (n = 20)	50.0 % (n = 10)	50.0 % (n = 30)	52.1 % (n = 74)
T3	13.0 % (n = 3)	15.4 % (n = 6)	25.0 % (n = 5)	26.7 % (n = 16)	21.1 % (n = 30)
T4	26.1 % (n = 6)	30.8 % (n = 12)	20.0 % (n = 4)	20.0 % (n = 12)	23.9 % (n = 34)
*N stage*					
N0	47.8 % (n = 11)	38.5 % (n = 15)	30.0 % (n = 6)	43.3 % (n = 26)	40.9 % (n = 58)
N1	30.4 % (n = 7)	12.8 % (n = 5)	35.0 % (n = 7)	13.3 % (n = 8)	19.0 % (n = 27)
N2	4.4 % (n = 1)	25.64 % (n = 10)	30.0 % (n = 6)	28.3 % (n = 17)	23.9 % (n = 34)
N3	17.4 % (n = 4)	23.1 % (n = 9)	5.0 % (n = 1)	15.0 % (n = 9)	16.2 % (n = 23)
*M stage*					
M0	100 % (n = 23)	97.4 % (n = 38)	100 % (n = 20)	100 % (n = 60)	99.3 % (n = 141)
M1	−	2.6 % (n = 1)	−	−	0.7 % (n = 1)
*GTV volume (cm^3^)*					
Median (min–max)	13.6 (2.3–84.9)	14.6 (1.5–97.2)	−	8.8 (0.7–85.1)	12.7 (0.7–97.2)

CRT was delivered according to local or national guidelines in each country, except for three patients in the UK ART cohort who were treated within the PLATO study (PersonaLising Anal cancer RadioTherapy dOse, ISRCTN88455282). Further details on CRT doses are given in Table 3*.*

**Table 3:** Summary of chemoradiotherapy received. Mitomycin (MMC); Intensity-modulated radiotherapy (IMRT); Volumetric Modulated Arc Therapy (VMAT); RapidArc Therapy (RA).

Characteristic		UK	Norway	Australia	Denmark	Combined
		(n = 23)	(n = 39)	(n = 20)	(n = 60)	(n = 142)
*Dose to primary tumour (Gy)*						
	Median	53.2	57.8	−	60.0	60.0
	(range)	(41.4–61.6)	(49.9–62.0)		(60.0–64.0)	(41.4–64.0)
*Chemotherapy regime: % (n)*						
	MMC/capecitabine	100 % (n = 23)	−	−	−	16.2 % (n = 23)
	MMC/5-fluorouracil	−	94.8 % (n = 37)	100 % (n = 20)	−	40.1 % (n = 57)
	Cisplatin/5-fluorouracil.	−	2.6 % (n = 1)	−	88.3 % (n = 53)	38.0 % (n = 54)
	No chemotherapy given	−	2.6 % (n = 1)	−	11.7 % (n = 7)	5.6 % (n = 8)
*Delivery technique: % (n)*						
	IMRT or VMAT	100 % (n = 23)	67.7 % (n = 26)	100 % (n = 20)	8.3 % (n = 5)	52.1 % (n = 72)
	RA	−	−	−	91.7 % (n = 55)	38.7 % (n = 55)
	3D conformal	−	33.3 % (n = 13)	−	−	9.2 % (n = 13)

The median follow-up (FU) from the start of CRT to the date of the last FU or death was 2.3 years for all patients combined (3.8 years for UK ART, 4.7 years for Norway, 2.9 years for Australia, and 1.3 years for Denmark).

Among all 142 patients, 11.3 % (n = 16) failed treatment locoregionally: 13.0 % (n = 3) of 23 in UK, 12.8 % (n = 5) of 39 in Norway, 15.0 % (n = 3) of 20 in Australia, and 9.8 % (n = 5) of 60 in Denmark. Among all 142 patients, 15.5 % (n = 22) had any treatment failure, including local, regional, or distant failure: 17.4 % (n = 4) of 23 in UK, 18.0 % (n = 7) of 39 in Norway, 25.0 % (n = 5) of 20 in Australia, and 10.0 % (n = 6) of 60 in Denmark.

### 3.1 ADC parameters and association with treatment failure

The median (range) ADCmean in the 1st and 2nd scans and the change between scans (ΔADCmean) for all patients were 900.0 × 10–6 mm^2^/s (199.3 to 1802.4), 1087.3 × 10–6 mm^2^/s (234.0 to 2230.2), and 22.8 % (−72.7 to 181.5), respectively. The full summary of ADC parameters for each centre and combined is available in Table A of supplementary material.

Table 4, Table 5 show the (trial-adjusted) odds of any and locoregional treatment failure, respectively and the AUC for each ADC parameter. No parameter was associated with locoregional or any treatment failure at baseline, mid-treatment, or as a percentage change between scans (all 95 % CIs included 1).

**Table 4:** Trial-adjusted Odds ratio (OR) and Area under the curve (AUC) for the association between Apparent diffusion coefficient (ADC) parameters and any treatment failure. Odds ratio (OR); Area under the curve (AUC); Apparent diffusion coefficient (ADC); Standard deviation (SD); Confidence interval (CI); Chemoradiotherapy (CRT).

ADC parameter	Baseline		Mid-CRT		Percentage change	
	OR (95 % CI)	AUC (95 % CI)	OR (95 % CI)	AUC (95 % CI)	OR (95 % CI)	AUC (95 % CI)
Mean	1.00 (1.00, 1.00)	0.623 (0.506, 0.740)	1.00 (1.00, 1.00)	0.642 (0.516, 0.768)	1.00 (0.99, 1.02)	0.552 (0.427, 0.677)
SD	1.00 (0.99, 1.01)	0.585 (0.453, 0.717)	1.00 (1.00, 1.01)	0.577 (0.447, 0.706)	1.00 (0.99, 1.01)	0.569 (0.445, 0.693)
Max	1.00 (1.00, 1.00)	0.608 (0.488, 0.727)	1.00 (1.00, 1.00)	0.563 (0.441, 0.686)	1.01 (0.99, 1.03)	0.583 (0.454, 0.713)
Skewness^1^	0.49 (0.22, 1.08)	0.652 (0.508, 0.796)	0.80 (0.37, 1.73)	0.550 (0.394, 0.705)	1.00 (1.00, 1.00)	0.562 (0.418, 0.705)
Kurtosis^1^	0.74 (0.50, 1.10)	0.613 (0.460, 0.765)	1.01 (0.73, 1.40)	0.541 (0.394, 0.689)	1.00 (1.00, 1.01)	0.546 (0.398, 0.694)

^1^These parameters were not available in the Denmark data, so Denmark was excluded from this analysis.

**Table 5:** Trial-adjusted Odds ratio (OR) and Area under the curve (AUC) for the association between Apparent diffusion coefficient (ADC) parameters and locoregional treatment failure. Odds ratio (OR); Area under the curve (AUC); Apparent diffusion coefficient (ADC); Standard deviation (SD); Confidence interval (CI); Chemoradiotherapy (CRT).

ADC parameter	Baseline		Mid-CRT		Percentage change	
	OR (95 % CI)	AUC (95 % CI)	OR (95 % CI)	AUC (95 % CI)	OR (95 % CI)	AUC (95 % CI)
Mean	1.00 (1.00, 1.00)	0.632 (0.492, 0.771)	1.00 (1.00, 1.00)	0.623 (0.466, 0.780)	1.00 (0.98, 1.01)	0.537 (0.370, 0.704)
SD	1.00 (0.99, 1.01)	0.575 (0.427, 0.723)	1.00 (0.99, 1.01)	0.544 (0.393, 0.696)	1.00 (0.99, 1.02)	0.524 (0.354, 0.693)
Max	1.00 (1.00, 1.00)	0.601 (0.443, 0.759)	1.00 (1.00, 1.00)	0.584 (0.423, 0.744)	1.00 (0.98, 1.02)	0.551 (0.389, 0.712)
Skewness^1^	0.42 (0.16, 1.09)	0.679 (0.512, 0.846)	0.63 (0.25, 1.61)	0.592 (0.404, 0.781)	1.00 (1.00, 1.00)	0.589 (0.412, 0.766)
Kurtosis^1^	0.77 (0.48, 1.24)	0.579 (0.417, 0.740)	1.08 (0.74, 1.56)	0.564 (0.404, 0.725)	1.00 (1.00, 1.01)	0.677 (0.507, 0.847)

^1^These parameters were not available in the Denmark data, so Denmark was excluded from this analysis.

There were 54 patients with a percentage change in ADCmean <20 %, of whom 18.5 % (n = 10) had a treatment failure anywhere, and 16.7 % (n = 9) failed locoregionally versus 88 patients with a change in ADCmean ≥ 20 %, of whom 13.6 % (n = 12) had a treatment failure anywhere, and 8.0 % (n = 7) failed locoregionally.

**Fig. 1:**
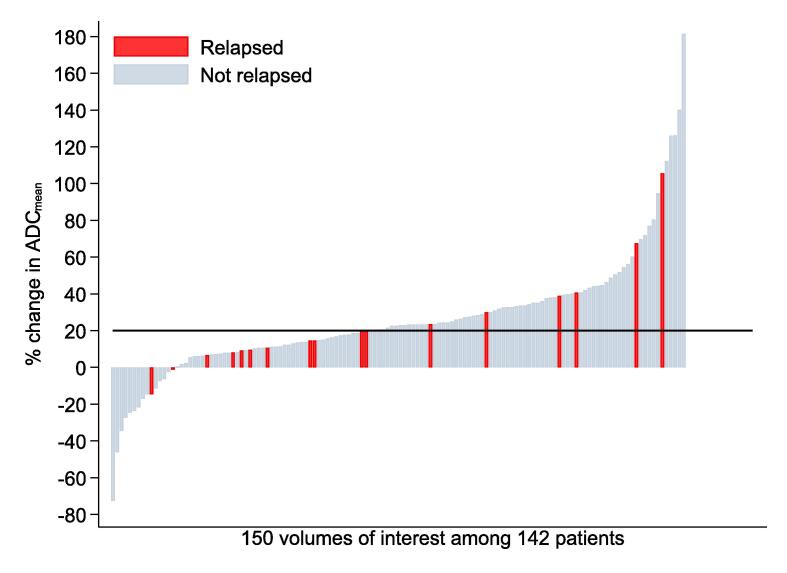
Waterfall plot of percentage change in ADCmean for locoregional failure. Apparent diffusion coefficient (ADC).

Fig. 1 illustrates the percentage change in ADCmean for locoregional failure using a waterfall plot. A waterfall plot of any failure is available in supplementary materials (Figure A).

The parameter ADCmin is not presented in this cohort as it represents the lowest single voxel value within the tumour volume. This parameter may represent a spurious value unless the voxels with non-physiological values are explicitly excluded in the post-processing of images. This was the case for some patients on inspection of the data, especially with data from one centre. Given the retrospective nature of this study and an inability to reanalyse ADCmin values, the decision was made to remove this parameter from the outcome analysis.

### 3.2 Subgroup analysis − primary tumours >5 cm

There were 69 patients with large primary tumours (>5 cm): 11 from the UK, 21 from Norway, 9 from Australia, and 28 from Denmark.

No parameter was statistically significantly associated with any treatment failure or locoregional treatment failure at baseline, mid-treatment, or as a percentage change between scans. *Tables B and C* in the supplementary material show the (trial-adjusted) odds of any and locoregional failure, respectively, and the AUC for each ADC parameter.

Among all failures, there were 21 patients with a percentage change in ADCmean <20 %, of whom 23.8 % (n = 5) failed treatment anywhere, and 23.8 % (n = 5) failed locoregionally versus 48 patients with a change in ADCmean ≥ 20 %, of whom 20.8 % (n = 10) failed anywhere, and 12.5 % (n = 6) failed locoregionally.

### 3.3 Correlate % change in ADC with % change in GTV

The correlation coefficient was −0.141 in this study, suggesting weak to no correlation between percentage change in ADCmean and percentage change in GTV. A figure illustrating the lack of correlation between percentage change in ADCmean and percentage change in GTV is available in supplementary materials (*Figure B*). Similarly, a correlation coefficient of −0.174 was obtained in the subgroup with large primary tumours (Figure C in supplementary material).

## 4 Discussion

We present an individual patient data *meta*-analysis (IPM) of patients undergoing CRT for SCCA, investigating DW-MRI as a potential early biomarker for locoregional and any failure. Unlike in two of the smaller trials, we found no association between locoregional or any treatment failure and any ADC parameter from paired DW-MRI at the outset and mid-treatment or the percentage change.

The primary endpoint of the UK ART trial continued to demonstrate a potential association with locoregional treatment failure. Patients who showed a change in ADCmean <20 % had twice the rate of failed treatment compared to those with ADCmean ≥ 20 %, both in the entire cohort and the smaller subset of large (>5 cm) primary tumours.

It is not a great surprise we have different results in the publications of the original studies, than found in the IPM. This is in keeping with the contradicting evidence to date on DW-MRI as a biomarker in SCC tumours of all sites. There are publications suggesting ADC histogram parameters mean, kurtosis, and skewness were not prognostic of outcome [23], [24], [25]. However, a positive correlation for some parameters has been reported in others [23], [25]. A few small studies [11], [22], [23] have also identified a percentage change that differentiates between good and bad outcomes, namely cut-offs of 14 %, 15.5 %, and 18 %. Small single-centre series, where data mining of multiple parameters is performed without a clear preplanned analysis, will eventually highlight a correlation that is not statistically or clinically robust. Consideration must be given to how the investigation of DW-MRI as a potential biomarker is done moving forward, so we avoid these studies that offer little to our understanding.

There are explanations for different results, such as different anatomical localisations of the tumours, motion artefacts or treatment differences. However, there are wider considerations in the investigation of DW-MRI as a biomarker. Differences in hardware, imaging protocols, acquisition parameters [26], software and methods for data-analysis [27] can all have a significant impact on results. There were some differences among the centres in the DWI acquisition parameters (Table 1). We recognise that homogenising and optimising these acquisition parameters are relevant. However, the echo times and repetition times used in the studies are not expected to give a substantial contribution to variations in ADC, as indicated by Celik [28]. Different sets of b-values have been employed at each centre, which may have led to some systematic differences [7]. In our analyses, we focused on the relative change in ADC parameters; thus, systematic variations between centres were not expected to influence the results to a great extent.

There are different methods of investigating DW-MRI as a biomarker. The gold standard is within prospective clinical trials, likely as an adjunct to a clinical question. While biological biomarkers are often integrated into planned large trials in rare tumour types, it is rare for radiological biomarkers to be incorporated. While there are guidelines on how the differences of DW-MRI at different centres may be minimised in a trial [29], there will continue to be different hardware used, possibly different processing software and the VOI are likely to be drawn by radiation oncologists at local centres., Therefore, a degree of heterogeneity and potential “noise” will exist even in the prospective clinical trial. We have demonstrated feasibility of an IPM in accordance with the PRISMA-IPD reporting guidelines for individual patient data *meta*-analysis [17]. Our method of using the analysed individual patient data, which is not available in the summary results presented in the individual trials, is in keeping with pharmaceutical trials; where the collected data is analysed, rather than any attempt at revisiting recruiting centres to re-extract the data prior to analysis. A larger funded piece of work could have centralised the original MRI scans, with centralised delineation and analysis; however, this was prohibitively expensive for the purposes of this paper. While this strategy would homogenise the software for analysis, the issues of different hardware, acquisition parameters, populations, investigations and treatments would remain. For these reasons, while we acknowledge the “noise”, it is present to a degree in all forms of radiological biomarker research. We have made all efforts to minimise this by following the PRIMSA-IPD guidelines and have avoided data manipulation or data mining by having a clear statistical analysis at outset [17]. Acknowledging the challenges of investigating DW-MRI as a biomarker, we believe this IPM contributes to the growing literature-base and, in its consideration, raises important points about the future of research into radiological biomarkers.

While DW-MRI alone, at these timeframes, probably is not a robust biomarker in anal cancer, there remain other potential imaging biomarkers of interest. Routinely performed MRI examinations usually consist of different MR sequences, each providing different imaging information. There have been correlations identified between outcome and different first- or higher-order texture parameters on T2-weighted MRI scans [30], [31], [32], and with tumour size-based parameters [11], [15], [33]. Studies investigating the prognostic role of FDG-PET in SCCA have assessed pre, post and during CRT images and identified potential correlations [34], [35], [36].

There has also been an explosion of interest in biological prognostic biomarkers since HPV16 and p16 were both found to be independently prognostic of overall survival [37], [38], [39]. Further work demonstrated tumour infiltrating lymphocytes (TIL’s) can complement the prognostic value of HPV status [37], [38]. More recently, much excitement surrounds the role of circulating tumour DNA (ctDNA). [40], [41].

Moving forward, all potential biomarkers should be analysed together in large studies in order to robustly investigate biomarkers individually and, importantly, in combination. It has been proposed to use several potential biomarkers to complement each other within a decision-making model which may be a more realistic aim than any one individual biomarker to inform decisions [42].

In summary, our study suggests that ADC histogram changes within the initial three weeks of CRT for SCCA are not associated with the odds of any treatment failure. IPM is a feasible method of increasing patient numbers and number of events in biomarker studies with some limitations. Incorporating radiological biomarkers into multicentre trials is the optimal way of identifying a cohort of complementary radiological and biological biomarkers to individualise treatment from diagnosis through radical treatment to recurrence and beyond [42].

## CRediT authorship contribution statement

**Bettina A. Hanekamp:** Conceptualization, Methodology, Data curation, Writing – original draft, Visualization, Project administration. **Pradeep S. Virdee:** Formal analysis, Data curation, Investigation, Software, Writing – original draft, Visualization. **Vicky Goh:** Conceptualization, Methodology, Investigation, Writing – review & editing, Validation. **Michael Jones:** Conceptualization, Methodology, Investigation, Data curation. **Rasmus Hvass Hansen:** Data curation, Writing – review & editing. **Helle Hjorth Johannesen:** Data curation. **Anselm Schulz:** Data curation, Visualization, Resources. **Eva Serup-Hansen:** Data curation, Writing – review & editing. **Marianne G. Guren:** Conceptualization, Methodology, Investigation, Data curation, Writing – review & editing, Supervision, Validation. **Rebecca Muirhead:** Supervision, Project administration, Conceptualization, Methodology, Investigation, Writing – original draft, Writing – review & editing, Validation.

## Declaration of competing interest

The authors declare that they have no known competing financial interests or personal relationships that could have appeared to influence the work reported in this paper.
