# Supplementary material for: Diffusion-weighted magnetic resonance imaging as an early prognostic marker of chemoradiotherapy response in squamous cell carcinoma of the anus: An individual patient data meta-analysis
Source: Phys Imaging Radiat Oncol. 2024 Jul 31;31:100618. doi: 10.1016/j.phro.2024.100618 (PMC11345337; doi:10.1016/j.phro.2024.100618)
Supplement: Supplementary Data 1 [file mmc1.pdf]

Supplementary Material

Table A:

ADC parameter	Study	Mean	SD	Median	Min	Max
Mean (scan 1)	UK	920.5	177.6	941.6	486.9	1230.1
	Norway	1007.5	111.1	1000.0	810.0	1250.0
	Australia	824.1	159.3	821.7	445.7	1193.7
	Denmark	784.2	318.7	713.7	199.3	1802.4
	Total	884.0	242.3	900.0	199.3	1802.4
Mean (scan 2)	UK	1146.4	184.5	1140.9	687.9	1600.4
	Norway	1211.2	123.9	1210.0	1000.0	1540.0
	Australia	893.8	252.9	980.6	234.0	1165.3
	Denmark	978.4	361.5	912.3	269.3	2230.2
	Total	1073.4	280.7	1087.3	234.0	2230.2
% change in mean	UK	28.9	34.3	20.7	-7.5	140.1
	Norway	20.8	11.5	19.3	2.4	44.8
	Australia	11.8	39.5	19.1	-72.7	126.3
	Denmark	36.0	40.8	35.2	-34.6	181.5
	Total	26.1	33.1	22.8	-72.7	181.5
SD (scan 1)	UK	301.7	101.2	290.1	176.1	609.3
	Norway	241.6	55.6	236.0	121.0	372.0
	Australia	229.2	83.6	214.4	94.4	377.8
	Denmark	222.5	75.5	209.6	108.0	451.9
	Total	243.9	81.1	233.0	94.4	609.3
SD (scan 2)	UK	267.3	74.6	257.6	124.3	422.3
	Norway	242.4	78.7	228.0	105.0	542.0
	Australia	236.8	84.3	222.2	113.9	405.1
	Denmark	223.8	80.4	200.6	99.0	482.7
	Total	240.0	80.0	227.2	99.0	542.0
% change in SD	UK	-6.9	26.9	-4.7	-58.3	72.6
	Norway	1.0	23.1	-2.3	-39.5	48.9
	Australia	10.5	43.3	7.8	-49.0	161.7
	Denmark	9.7	42.1	3.0	-49.4	131.7
	Total	3.7	34.5	-1.0	-58.3	161.7
Max (scan 1)	UK	1917.6	307.1	1905.0	1478.0	2637.0
	Norway	1945.0	357.6	1930.0	1280.0	2860.0
	Australia	1657.3	300.2	1631.0	1131.0	2418.0
	Denmark	1333.7	491.6	1230.8	532.6	3040.0
	Total	1675.6	481.5	1631.0	532.6	3040.0
Max (scan 2)	UK	2020.8	377.4	1983.5	1405.0	2937.0
	Norway	1998.4	336.0	1915.0	1460.0	2770.0
	Australia	1650.8	361.0	1693.0	774.0	2101.0
	Denmark	1446.0	487.8	1356.6	693.7	3161.9
	Total	1764.7	476.4	1769.5	693.7	3161.9
% change in max	UK	6.0	15.9	1.1	-19.3	48.5

	Norway	3.7	11.3	4.3	-19.1	35.1
	Australia	-0.3	17.1	-4.3	-31.6	33.0
	Denmark	16.3	33.1	9.0	-37.6	101.9
	Total	7.7	22.9	4.8	-37.6	101.9
Skewness (scan 1)[1]	UK	0.3	0.6	0.4	-1.1	1.5
	Norway	0.8	0.6	0.8	-0.7	2.0
	Australia	0.5	0.9	0.6	-1.2	2.0
	Denmark					
	Total	0.6	0.7	0.6	-1.2	2.0
Skewness (scan 2)[1]	UK	0.0	0.6	0.0	-0.9	2.1
	Norway	0.4	0.6	0.4	-1.3	1.4
	Australia	0.2	0.8	0.4	-0.9	2.0
	Denmark					
	Total	0.2	0.7	0.3	-1.3	2.1
% change in skew[1]	UK	-103.5	339.9	-72.9	-1133.3	866.7
	Norway	-9.4	232.3	-34.4	-450.0	1266.7
	Australia	-51.4	212.2	-15.3	-840.0	178.6
	Denmark					
	Total	-45.9	264.1	-45.0	-1133.3	1266.7
Kurtosis (scan 1)[1]	UK	3.3	1.2	2.9	2.2	6.2
	Norway	4.2	1.6	3.7	2.4	9.3
	Australia	1.3	1.9	1.2	-1.2	5.7
	Denmark					
	Total	3.3	1.9	3.3	-1.2	9.3
Kurtosis (scan 2)[1]	UK	3.5	1.4	3.5	0.0	7.0
	Norway	3.9	1.2	3.6	2.1	7.4
	Australia	1.1	2.2	0.7	-1.1	8.2
	Denmark					
	Total	3.2	1.9	3.2	-1.1	8.2
% change in kurtosis[1]	UK	8.2	42.7	0.3	-98.8	94.1
	Norway	1.7	34.7	2.5	-63.5	69.1
	Australia	38.5	279.5	-49.3	-173.3	825.0
	Denmark					
	Total	11.8	134.1	-4.0	-173.3	825.0

[1]These parameters were not available in the Denmark data, so Denmark was excluded here

Table supplementary A: Summary of ADC ($x10^{-6}$ mm^2/s) parameters. Apparent diffusion coefficient (ADC); Standard deviation (SD);

Table B:

ADC parameter	Baseline		Mid-CRT		Percentage change	
	OR (95% CI)	AUC (95% CI)	OR (95% CI)	AUC (95% CI)	OR (95% CI)	AUC (95% CI)
Mean	1.00 (0.99, 1.00)	0.712 (0.570, 0.854)	1.00 (0.99, 1.00)	0.731 (0.584, 0.878)	1.00 (0.98, 1.02)	0.656 (0.494, 0.818)
SD	1.00 (0.99, 1.00)	0.706 (0.556, 0.856)	1.00 (0.99, 1.00)	0.681 (0.535, 0.828)	1.01 (0.99, 1.02)	0.670 (0.512, 0.829)
Max	1.00 (1.00, 1.00)	0.763 (0.606, 0.920)	1.00 (1.00, 1.00)	0.733 (0.589, 0.878)	1.01 (0.98, 1.04)	0.663 (0.495, 0.831)
Skewness[1]	0.28 (0.07, 1.08)	0.780 (0.618, 0.941)	0.46 (0.16, 1.37)	0.747 (0.568, 0.925)	1.00 (0.99, 1.00)	0.694 (0.524, 0.864)
Kurtosis[1]	0.78 (0.45, 1.35)	0.686 (0.507, 0.864)	1.02 (0.70, 1.49)	0.725 (0.561, 0.888)	1.00 (1.00, 1.00)	0.719 (0.555, 0.883)

[1]These parameters were not available in the Denmark data, so Denmark was excluded from this analysis

Table supplementary B: Trial-adjusted Odds ratio (OR) and Area under the curve (AUC) for the association between Apparent diffusion coefficient (ADC) parameters and **any failure** in those with *big primary tumours (>5 cm)*. Odds ratio (OR); Area under the curve (AUC); Apparent diffusion coefficient (ADC); Standard deviation (SD); Confidence interval (CI); Chemoradiotherapy (CRT)

Table C:

ADC parameter	Baseline		Mid-CRT		Change	
	OR (95% CI)	AUC (95% CI)	OR (95% CI)	AUC (95% CI)	OR (95% CI)	AUC (95% CI)
Mean	1.00 (0.99, 1.00)	0.674 (0.505, 0.843)	1.00 (0.99, 1.00)	0.685 (0.500, 0.869)	0.99 (0.97, 1.01)	0.583 (0.379, 0.787)
SD	1.00 (0.99, 1.00)	0.617 (0.434, 0.800)	1.00 (0.99, 1.01)	0.573 (0.380, 0.766)	1.01 (0.99, 1.03)	0.635 (0.425, 0.844)
Max	1.00 (1.00, 1.00)	0.709 (0.512, 0.906)	1.00 (1.00, 1.00)	0.676 (0.504, 0.847)	1.00 (0.97, 1.03)	0.582 (0.385, 0.779)
Skewness[1]	0.30 (0.07, 1.26)	0.753 (0.567, 0.940)	0.44 (0.13, 1.45)	0.691 (0.477, 0.905)	1.00 (0.99, 1.00)	0.670 (0.468, 0.872)
Kurtosis[1]	0.78 (0.43, 1.43)	0.632 (0.438, 0.826)	1.02 (0.67, 1.55)	0.628 (0.424, 0.833)	1.00 (1.00, 1.01)	0.674 (0.479, 0.868)

[1]These parameters were not available in the Denmark data, so Denmark was excluded from this analysis

Table supplementary C: Trial-adjusted Odds ratio (OR) and Area under the curve (AUC) for the association between Apparent diffusion coefficient (ADC) parameters and **locoregional failure** in those *with big primary tumours (>5 cm).* Odds ratio (OR); Area under the curve (AUC); Apparent diffusion coefficient (ADC); Standard deviation (SD); Confidence interval (CI); Chemoradiotherapy (CRT)

Figure A:

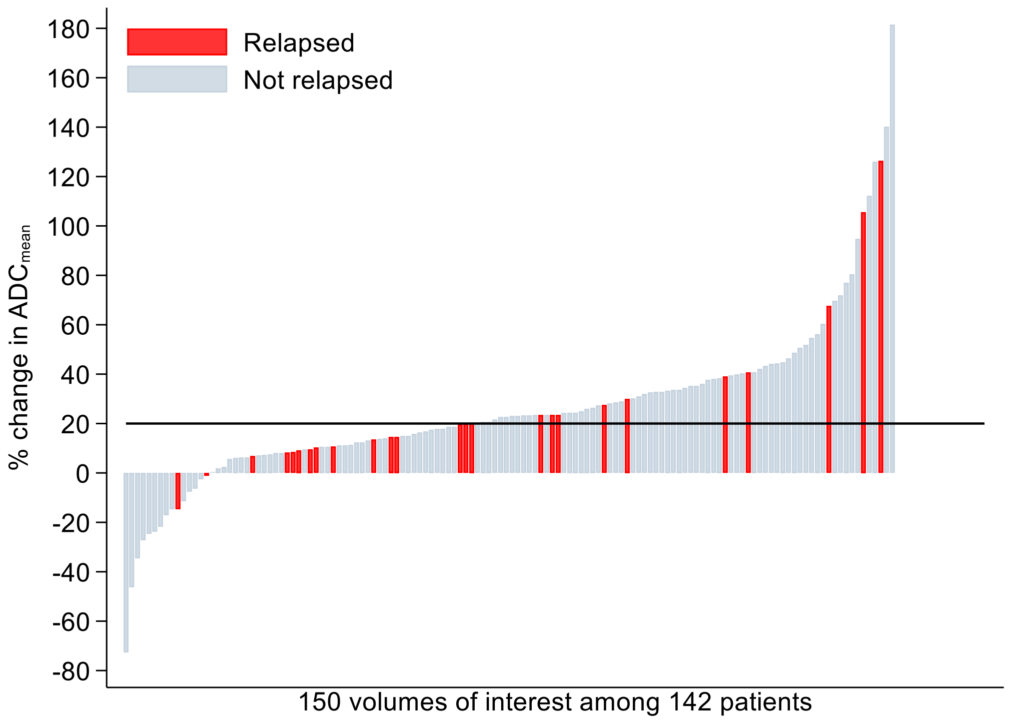

Figure supplementary A: Waterfall plot of percentage change in ADCmean for **any failure.** Apparent diffusion coefficient (ADC)

Figure B:

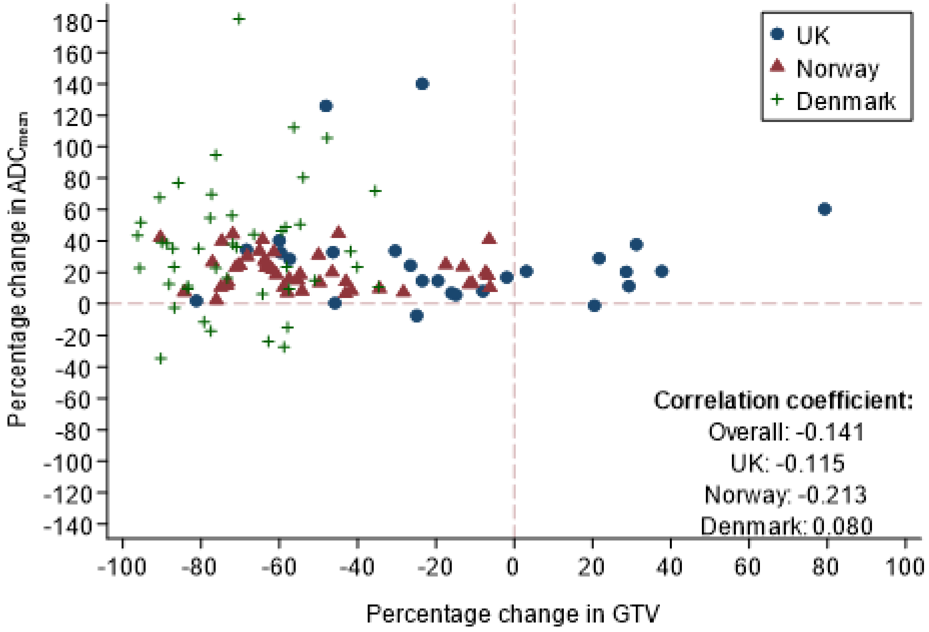

Figure supplementary B: Spearman correlation between percentage change in ADCmean and percentage change in GTV between the scan at baseline and mid-CRT. Apparent diffusion coefficient (ADC); Gross tumour volume (GTV)

Figure C:

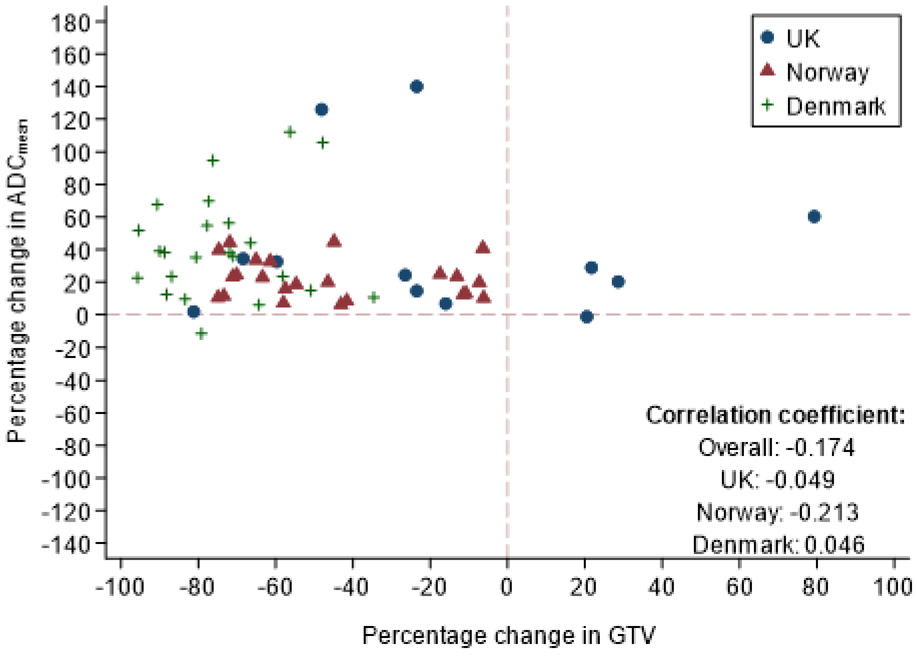

Figure supplementary C: Spearman correlation between percentage change in ADCmean and percentage change in GTV between the scan at baseline and mid-CRT in those with *big primary tumours.* Apparent diffusion coefficient (ADC); Gross tumour volume (GTV)
